# Non-Contact and Wireless Wearable Technologies for Neonatal Vital Sign Monitoring in the Delivery Room—A Scoping Review

**DOI:** 10.3390/children13081025

**Published:** 2026-08-01

**Authors:** Eva Sutera, Alyssa Maximov, Vívian Mara Gonçalves de Oliveira Azevedo, Wissam Shalish, Robert Kearney, Guilherme M. Sant’Anna

**Affiliations:** 1 Faculty of Medicine and Health Science, McGill University, Montreal, QC H3A 0G4, Canada; eva.sutera@mail.mcgill.ca; 2 Research Institute of the McGill University Health Center, Montreal, QC H4A 3J1, Canada; alyssa.maximov@muhc.mcgill.ca (A.M.); wissam.shalish@mcgill.ca (W.S.); robert.kearney@mcgill.ca (R.K.); 3 Faculty of Physical Education and Physiotherapy, Federal University of Uberlândia (FAEFI-UFU), Rua Benjamin Constant, 1286, Bairro Aparecida, Uberlândia 38400-678, MG, Brazil; vivian.azevedo@ufu.br; 4 Neonatal Division, McGill University Health Center, Montreal, QC H4A 3H9, Canada

**Keywords:** wearable devices, non-contact, vital sign monitoring, wireless technology, delivery room, newborn

## Abstract

**Highlights:**

**What are the main findings?**

- Heart rate is monitored in 100% of studies.
- Most devices capture only one vital sign.

**What are the implications of the main findings?**

- Wireless systems are a promising direction for vital sign monitoring in delivery room care, but larger and more standardized studies are required to confirm accuracy, safety, and feasibility before clinical adoption.
- The predominance of heart-rate-only, short-duration monitoring implies that current devices do not yet provide comprehensive newborn surveillance, so future development should focus on multi-vital-sign systems that can better fit the delivery room environment and reduce reliance on cumbersome wired monitors.

**Abstract:**

Background: Vital sign monitoring immediately after birth is a necessity to aid the transition to life, especially in the context of resuscitation. Current monitoring systems pose challenges due to their wired nature. This scoping review aimed to identify and describe non-contact and wireless vital sign monitoring technologies used immediately after birth and summarize their capabilities and any research gaps to better understand the current state of wireless monitoring in the delivery room setting. Methods: The review followed the JBI 9-step framework and PRISMA-ScR guidance. Searches were conducted in Medline, Embase, Scopus, Web of Science, CINAHL, and Cochrane for studies published from 1 January 2015 to 1 October 2025, with additional reference screening of included articles. Study and device characteristics and study outcomes (i.e., accuracy, feasibility, safety) were collected via a data collection form and analyzed and presented by descriptive methods. Results: The search yielded 59,220 records; after duplicate removal and screening, seven full articles were included, one additional article was added through cross-reference screening. All eight studies were conducted in hospital delivery rooms, mostly as prospective observational designs, involving newborns of various gestational ages and weights, with a median of 29 [IQR:35] participants per study. All eight studies evaluated wearable devices. Heart rate was the most commonly monitored vital sign, and Bluetooth was the main data transfer method. Recording periods were mostly under 10 min. Conclusions: New monitoring technologies used immediately after birth are emerging, but data remains preliminary and limited by small studies with short recording periods. Future work should emphasize standardized device placement, larger samples with longer monitoring periods, rigorous accuracy and safety evaluation, and devices that can capture multiple vital signs reliably.

## 1. Introduction

Decreasing neonatal mortality remains a critical global health challenge. The Sustainable Development Goals aim to reduce avoidable newborn deaths to ≤12 per 1000 live births by 2030 [1,2]. A reduction from 18 to 12 per 1000 could save nearly 1 million lives annually, with a further drop to 5 per 1000 potentially saving another million. Of the 2.3 million annual neonatal deaths, over 80% are of a low birthweight, and more than half are preterm infants [2,3].

Since 1987, the Neonatal Resuscitation Program has established that timely and effective resuscitation immediately after delivery improves survival and important outcomes. While most newborns breathe independently, about 10% require medical assistance and 1% require intensive resuscitation [4,5]. For these situations, resuscitation guidelines emphasize continuous monitoring of vital signs. However, the delivery room is a highly specialized environment demanding accurate, rapid, and reliable monitoring systems suited for both term and preterm infants [6].

Heart rate, best measured by electrocardiography (ECG), provides the fastest and most accurate assessment of cardiorespiratory status and response to interventions. Pulse oximetry, although essential, can underestimate or overestimate heart rate in the first minutes after birth [7,8]. Skin temperature must be continuously maintained between 36.5 °C and 37.5 °C, as deviations increase the risk of morbidity and mortality in late preterm and low-birth-weight infants [9,10]. Oxygen saturation should be monitored to guide oxygen therapy and maintain normal ranges within the first 10 min of life, avoiding both hypoxia and hyperoxia.

In the delivery room, standard wired monitors can provide delayed or unreliable data in the first critical minutes of life due to the fragility and wet skin of newborns that complicate proper electrode placement [11,12,13,14,15]. Wired sensors can easily tangle, disconnect, or obstruct procedures [11,16]. Consequently, emerging non-contact and wireless technologies, such as diaphragm activity sensors, camera-based systems, lasers, and wireless electrodes, are being explored to enhance vital sign monitoring immediately after birth [17,18,19].

This scoping review aims to identify and describe non-contact or wireless vital sign monitoring technologies used immediately after birth, highlighting current advancements, reliability, and potential for neonatal care innovation.

## 2. Materials and Methods

This scoping review followed the guidelines of the 9-step framework from the JBI methodology [20]. It has been registered on Open Science Framework (DOI: 10.17605/OSF.IO/NDWFA), and the reporting uses the PRISMA for Scoping Reviews (PRISMA-ScR) guidelines [21].

### 2.1. Search Strategy

The search strategy was developed with help from a McGill University Health Center librarian. Literature searches were performed in 6 databases: Medline, Embase, Scopus, Web of Science, CINAHL, and Cochrane. The search strategy, including all identified keywords and index terms, was adapted for each database, and the information source was included. The reference lists of all included sources were screened for additional studies. Sources of unpublished studies/gray literature searches included trial registers and websites of manufacturers of identified devices. Key search terms are provided in Supplementary Table S1. Due to the fast-paced and always-evolving nature of technologies, results were limited to articles published between 1 January 2015 and 1 October 2025. There were no language restrictions applied.

### 2.2. Inclusion/Exclusion Criteria

Both wireless wearable and non-contact monitoring technologies were considered eligible because the review was designed around the shared clinical objective of enabling cable-free vital sign monitoring in the delivery room. Although these technologies differ in sensing principles, physical interaction with the infant, and stage of technological development, both aim to overcome challenges associated with conventional wired monitoring systems, including delays in signal acquisition, interference with clinical procedures, limitations during delayed cord clamping, and restrictions on parent–infant contact. Given their common implementation goal in the delivery room environment, both technology categories were considered within the same evidence map while being analyzed according to their specific characteristics.

Publications included had to contain “delivery room, delivery room simulations, or technology development intended for the delivery room”, with “newborn patients or manikins”, and “wireless or non-contact technology for continuous vital sign monitoring”. Detailed inclusion and exclusion criteria used to select articles are summarized in Supplementary Table S2.

### 2.3. Studies Selection

Results were exported into Covidence (Veritas Health Innovation, Melbourne, Australia), and all duplicates were automatically removed. Using the predetermined eligibility criteria, all articles were screened by title and abstract first; the remaining articles underwent a full-text review. All articles were screened by 2 of the 3 independent reviewers (ES, AM, VOA). Any disagreements between reviewers at each stage of the selection process were resolved through a discussion or by the third reviewer.

### 2.4. Data Collection

A data collection form (Supplementary Document S1) was created to extract all relevant information from the final articles. The form was incorporated into Covidence, and the data extraction process for each selected article was done by 2 independent reviewers (ES, AM, VOA). Any conflict between the reviewers was resolved through a discussion or by a third reviewer. The following information was collected: authors, year of publication, study design, study aim, country of study site, number of sites, study setting, number of participants, participant characteristics, location and means of device placement, vital signals obtained by wireless device, number of wireless devices used, duration of monitoring period, reference sensors used, and outcomes and/or findings of the studies. Although data was collected, for the purpose of this review, the quality of the articles was not evaluated as the main goal of this scoping review was to describe the range of the research involving wireless or non-contact monitoring technology for newborns in the delivery room setting.

### 2.5. Data Analysis

Data analysis followed a descriptive approach consistent with the scoping review methodology. After charting the extracted data, studies were organized by key variables such as study location, population, wireless device type, vital signs measured, outcomes, and methodological characteristics. The data was analyzed with basic descriptive statistics and frequency counts and presented in tables and figures to draw a conclusion on gaps and patterns in the current literature on this topic.

## 3. Results

**Figure 1:**
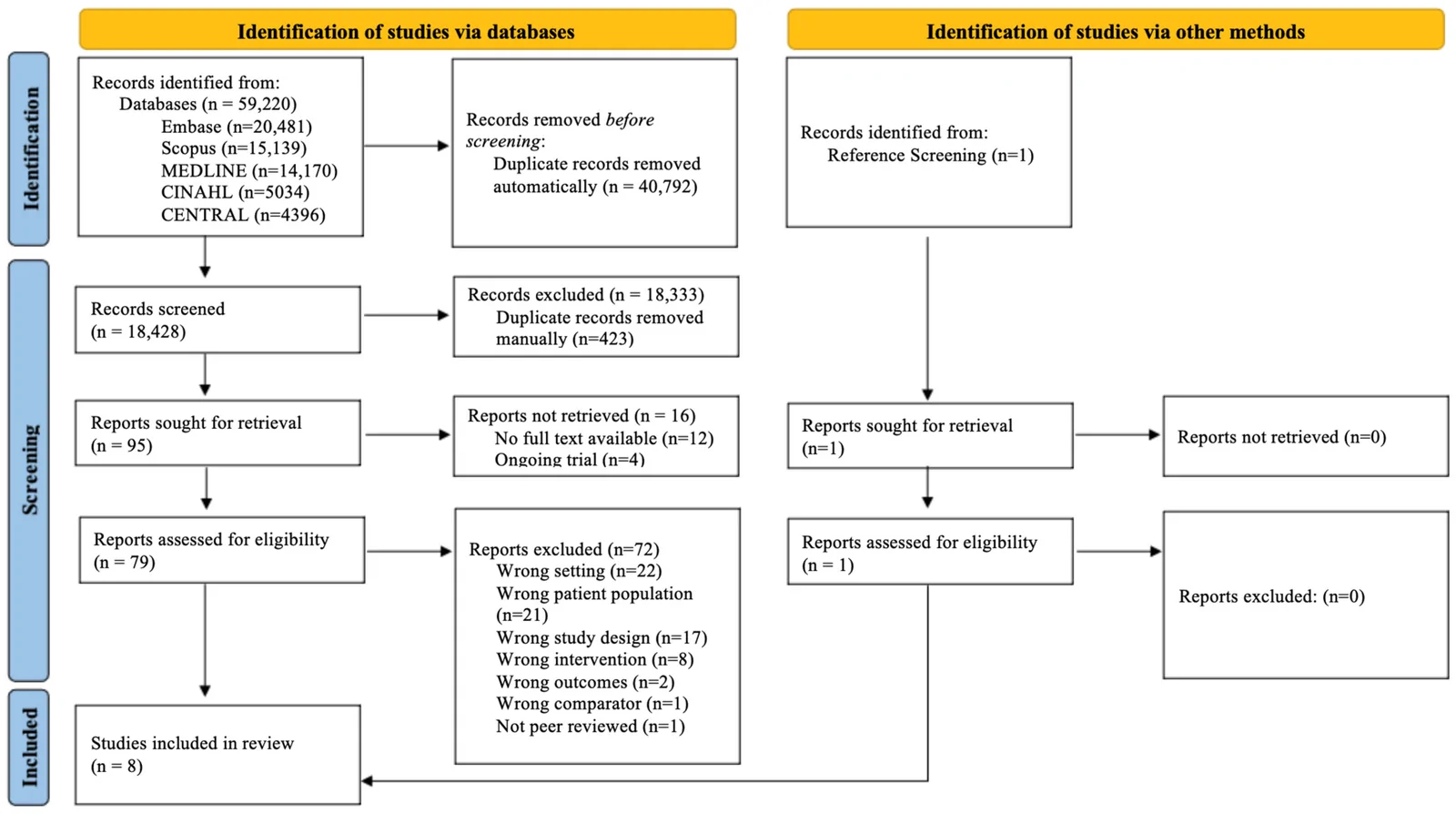
PRISMA flow diagram for assessment of search results.

The search yielded 59,220 articles that were imported into Covidence; 40,792 duplicates were automatically identified and removed, and 423 were manually identified and removed. 18,005 articles were screened by title and abstract and 95 underwent full-text review, leaving 7 full articles to be analyzed and included [22,23,24,25,26,27,28] (See Figure 1). An additional article [29] was found externally to the database searches by manual reference list screening of the included articles and was added to this scoping review as it fit the criteria of inclusion, bringing the total included articles to 8.

### 3.1. Demographics

**Figure 2:**
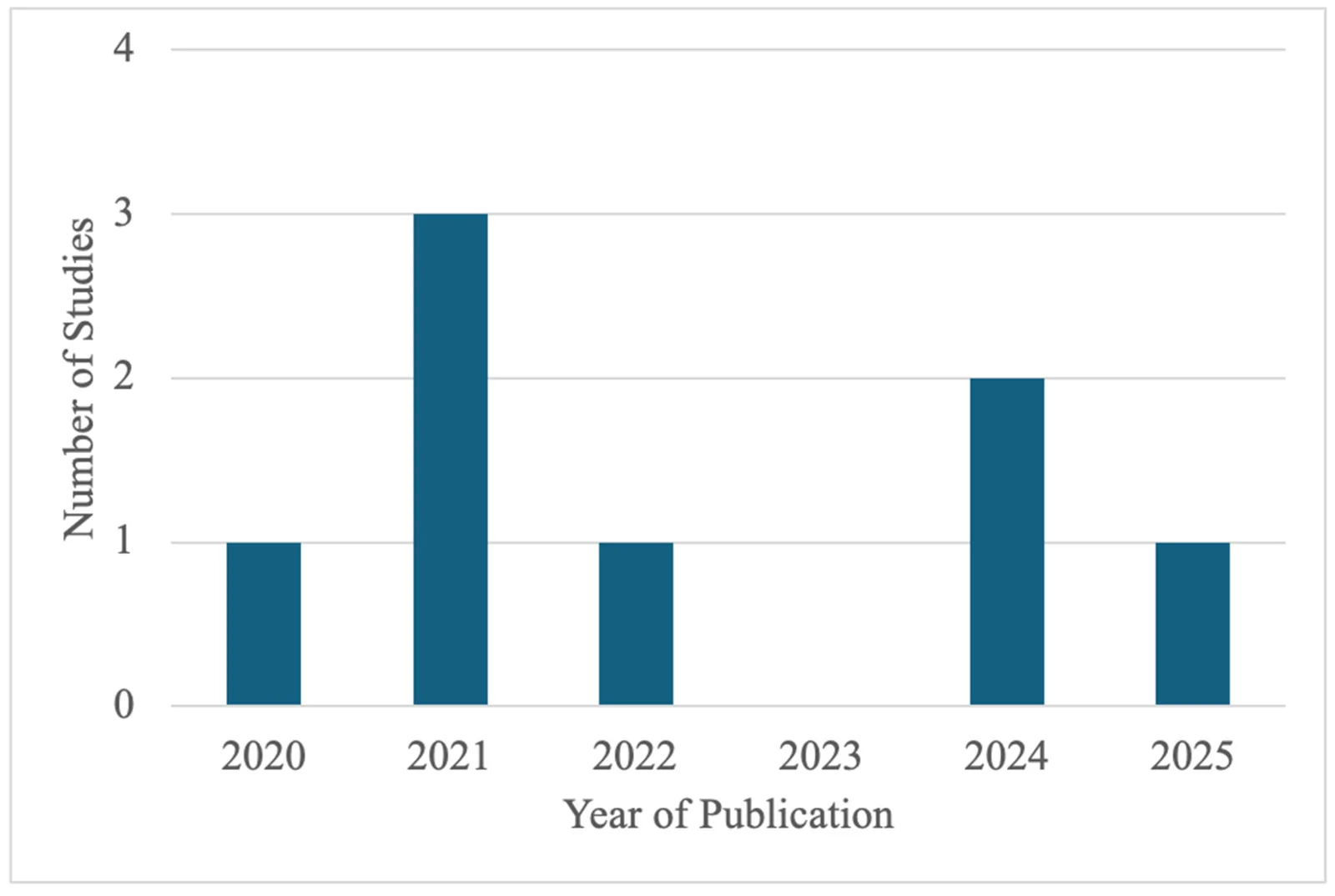
Year of publication per study for all included articles.

Of the eight included articles, studies were performed in: the United States of America = 2 (25%) [22,23], United Kingdom = 3 (37.5%) [24,25,26], Italy = 1 (12.5%) [27], Norway = 1 (12.5%) [28], and Netherlands = 1 (12.5%) [29]. Types of study were as follows: prospective observational studies *n* = 6 (75%) [22,23,24,26,27,29], prospective mixed-methods observational study = 1 (12.5%) [25], and prospective descriptive study = 1 (12.5%) [28]. All eight articles were published in the last 5 years, with more than half (*n* = 5, 62.5%) [23,24,27,28,29] being published before 2024 (See Figure 2 below). All articles were published in different journals, with only three (37.5%) [24,27,29] journals overlapping, see Supplementary Table S3.

### 3.2. Study Methodologies

#### 3.2.1. Study Site

All eight studies were conducted in the delivery room of hospitals [22,23,24,25,26,27,28,29]. Only one (12.5%) [26] study had two sites. Details of study methodologies can be found in Table 1.

#### 3.2.2. Population

The median number of participants across the eight studies was 29 [IQR: 35]. Seven of the eight studies included term infants [23,24,25,26,27,28,29], and some included preterm infants [22,23,25,28,29]. The frequency of each infant age group is available in Table 1.

#### 3.2.3. Devices

The articles included tested novel technologies and existing technologies in a new clinical setting, the delivery room. All studies used a reference technology that was the gold standard as a comparator to the device being evaluated. All articles described wearable devices. The NeoBeat (Laerdal, Stavanger, Norway) device was the most common existing device tested (*n* = 4, 50%) [22,23,28,29], and other studies tested different novel wearables, such as baby hats (*n* = 3, 37.5%) [24,25,26] and a jacket (*n* = 1, 12.5%) [27]. A summary of devices can be found in Table 2 below.

Duration of monitoring was extracted from each article and defined as the longest continuous recording or at the longest timestamp increment period that the wearable device was used. The reported monitoring durations varied within the studies: four (50%) [22,23,28,29] studies measured vital signs for only the first few minutes after birth and were resuscitation-focused recordings lasting less than 10 min, other studies obtained paired measurements at several time points extending up to 20 min [24] and 120 min after birth [27], one (12.5%) [26] study recorded the first 10 min of life not during resuscitations, and one (12.5%) [25] reported device was used across delivery room stays without a precise maximum duration, so it was classified as “unspecified.” In total, half of the studies described monitoring periods of less than 10 min, making the <10 min interval the most common duration reported across the articles. The frequency of monitoring periods can be found in Table 1.

#### 3.2.4. Outcomes

The outcomes of each study were separated into three categories: accuracy, safety, and feasibility. Device accuracy was assessed by agreement and correlation when compared to the wired standard system; feasibility was assessed by placement time of the device, signal acquisition time, and ability to obtain continuous data; and safety was assessed by adverse events related to wireless devices such as skin irritations and discomfort. Accuracy was most commonly reported in seven (87.5%) [22,23,24,26,27,28,29] studies, feasibility in six (75%) [22,23,24,25,26,28], and safety in only two (25%) [23,28]. Basic descriptives (mean, median, mode, min, max, inter quartile range, standard deviation, variance) were used in all eight studies (Table 1). One (12.5%) [25] study additionally included qualitative data from surveys that were given to the healthcare professionals and parents for feedback on the device being tested. All investigations aimed to validate the wireless signal against a reference wired monitoring system; the most commonly used was a three-lead ECG or a standard pulse oximeter, and only some studies (*n* = 5, 62.5%) [22,24,26,27,29] reported formal Bland–Altman agreement in their analysis.

### 3.3. Device Features

#### 3.3.1. Signals Monitored

Only signals recorded in each study were included in the analysis. Consequently, when a device is described as “monitoring” a signal, this refers to the signal that was reported, not to every signal that could theoretically be captured. The median number of signals reported per device was one [IQR:1]. The most prevalent number of vital signs (HR, temperature, and SpO2) monitored for each of the devices was also one.

The most common vital sign monitored was heart rate across all studies [22,23,24,25,26,27,28,29]; one (12.5%) [26] study also monitored SpO2 at the same time. None of the devices monitored temperature. It is worth mentioning that only one (12.5%) [26] out of the eight studies had a device that monitored more than one vital sign simultaneously.

Additionally, for the eight wearable technologies described in the selected articles, the acquisition method for each vital signal was recorded. For heart rate, the most frequently used technique was ECG (*n* = 5, 62.5%) [22,23,27,28,29], and the second was reflectance photoplethysmography (PPG) (*n* = 3, 37.5%) [24,25,26]. For SpO2, the device relied on multi-wavelength PPG (*n* = 1, 12.5%) [26]. More details can be found in Table 3 below.

Bluetooth was the predominant communication technology; seven of the eight devices transmitted data via Bluetooth (*n* = 7, 87.5%) [22,23,24,25,26,28,29]. Only one device used Wi-Fi (*n* = 1, 12.5%) [27] (Table 3).

#### 3.3.2. Placement

Placement of the wireless monitoring devices was grouped into five anatomical regions: neck, torso, head, upper limbs, lower limbs, and not on the body. All of the devices in the studies were placed once and only in one anatomical region. The torso and head were the most common, being used in five (*n* = 5, 62.5%) [22,23,27,28,29] and three (*n* = 3, 37.5%) [24,25,26] studies, respectively. The upper and lower limbs were not used by any study. Devices were further analyzed for placement according to each vital sign recorded; (a) HR = torso (*n* = 5, 62.5%) [22,23,27,28,29] and head (*n* = 3, 37.5%) [24,25,26], (b) SpO2 = head (*n* = 1, 100%) [26]. A more detailed distribution of device placement is shown in Table 4.

Securing mechanisms were classified into five categories as well: adhesives, band/elastic/strap, garment, non-contact, or not-secured device. Non-contact devices refer to technologies that do not touch the neonate’s body. Not-secured devices refer to technologies placed on the patient’s body but without the use of any measure to secure it to the body. Garment-based fixation was reported in four studies (*n* = 4, 50%) [24,25,26,27]. Not-secured devices were described in four studies (*n* = 4, 50%) [22,23,28,29]. Finally, adhesive-secured devices were not described in any wireless device used in the articles. More details can be found in Table 4.

## 4. Discussion

### 4.1. Study Characteristics

Our review identified eight wearable wireless monitoring systems, showing a slowly expanding research field, which are still under early clinical evaluation. The majority of the studies were prospective observational studies published between 2021 and 2025 involving a small number of participants with monitoring confined to the first minutes after birth or short bedside periods.

The population of focus for the eight clinical studies was newborns of various gestational ages and weights, with all studies conducted in delivery room settings but under different contexts, for example during skin-to-skin or resuscitation efforts. This emphasis reflects concerns about wired monitoring devices that impede clinical interventions and parent kangaroo care, increase infection risk and can potentially cause skin injury from the tangling and tugging of wires. Accordingly, many of the devices tested baby hats or fabric-integrated electrodes to minimize skin trauma and to be less invasive in this fragile population.

### 4.2. Device Characteristics

Novel monitoring technologies are increasingly improving and gaining traction. However, the majority of the devices analyzed in this scoping review only monitored one vital sign at a time, and none monitored temperature, an important physiological marker of a newborn’s wellbeing. No study reported continuous movement detection, although the ComfTech HOWDY jacket could theoretically record motion artefacts, aiding clinical diagnosis and decisions.

Bluetooth emerged as the predominant wireless data transfer method accounting for seven of the eight studies (*n* = 7, 87.5%) [22,23,24,25,26,28,29], while only one [27] relied on Wi-Fi. Bluetooth’s low power consumption and ease of miniaturization make it attractive for neonatal applications despite its limited range compared with Wi-Fi. The NeoBeat and SurePulse VS systems relied on Bluetooth for real-time data transmission to a smartphone or tablet. Similarly, the ComfTech HOWDY jacket uses Bluetooth to transmit physiological data from the wearable sensor to a bedside tablet, with Wi-Fi subsequently used for additional data transfer within the communication network. Therefore, Bluetooth was the primary technology used for real-time acquisition and transmission across most wireless monitoring systems included in this review. Bluetooth’s short-range, low-power profile enables miniaturized, battery-efficient units that are well suited to neonatal applications where prolonged skin contact and lightweight form factors are essential. However, its limited bandwidth and range make it less appropriate for high-volume data streams compared with Wi-Fi, which can support larger files but typically requires more power and a stable network connection. The prevalence of Bluetooth across the majority of devices reflects a trade-off that favors ease of integration and long-lasting operation over high-speed data transfer, aligning with the practical constraints of bedside neonatal monitoring.

### 4.3. Future Directions and Improvements

The studies included in this review demonstrated a new area of interest on the use of wireless monitoring immediately after birth. Technology in the modern world is constantly evolving and improving, and moving forward, it would be important to refine and tailor wireless monitoring devices to reflect the specific needs of newborns and the delivery room environment. The lack of studies with larger sample sizes and sophisticated data analysis highlights an area that needs more research to explore the potential benefits of wireless vital sign monitoring for newborns. While new technology may have the potential to improve clinical practices, it also poses some obstacles to overcome before it can be safely implemented. Some of the obstacles to pay attention to are device reliability, accuracy, safety, battery life, device cost, ease of use, and compliance with other equipment.

A key consideration for the adoption of wireless vital sign monitoring in the delivery room is the unique physiological and environmental context of neonatal resuscitation. Unlike the relatively controlled environment of the neonatal intensive care unit (NICU), the delivery room is characterized by rapid transitions, variable temperature and lighting, frequent caregiver movement, wet skin with vernix, and the urgent need for immediate clinical interventions. These conditions may pose challenges for contactless technologies such as camera- or radar-based systems, which can be susceptible to motion artefacts, occlusion by caregivers, and inconsistent signal acquisition during active resuscitation. Workflow factors further complicate monitoring implementation. For example, delayed cord clamping and the increasing emphasis on bedside stabilization often require monitoring systems that function reliably while maintaining infant proximity to the mother and without interfering with obstetrical care. Physiological considerations are also important when interpreting measurements from different body sites and sensing modalities. Forehead-based sensors may provide earlier detection of central perfusion than peripheral sites during the immediate postnatal transition, while PPG-based heart rate measurements may be less reliable during periods of low peripheral perfusion compared with ECG [12,26]. These differences may be particularly relevant when clinical decisions depend on reaching the 100-beats-per-minute threshold that guides neonatal resuscitation algorithms, as even small measurement discrepancies could influence decisions regarding positive-pressure ventilation or escalation of support [30].

Beyond technical performance, future studies should evaluate the safety, usability, and broader clinical implications of wireless monitoring technologies in this vulnerable population. Although wireless systems may reduce the clutter and handling associated with conventional wired devices, potential device-related adverse events—including sensor displacement, overheating, electromagnetic interference, and data transmission failures—require careful surveillance. Skin integrity represents another important consideration, particularly among preterm infants whose immature epidermis is highly susceptible to pressure injury and adhesive-related skin trauma [31]. Wireless sensors that minimize or eliminate adhesive exposure may offer advantages, but these benefits must be balanced against the need for secure attachment and reliable signal acquisition. In addition, wireless monitoring may facilitate emerging models of Family Integrated Care (FIC) by enabling continuous physiological surveillance while supporting earlier parental contact, skin-to-skin care, and reduced separation during stabilization. Such capabilities may also have implications for identifying physiological instability associated with sudden unexpected postnatal collapse (SUPC), a rare but potentially devastating event that often occurs during early parental contact. Whether wireless monitoring can enhance the early detection of cardiorespiratory deterioration while preserving the benefits of family-centered care remains an important area for future investigation.

Therefore, emerging research studies should focus on implementation of standardized methods for device placement, large sample sizes, longer monitoring periods in both delivery room resuscitation scenarios and skin-to-skin bonding, accuracy, safety, and feasibility analyses, as well as monitoring multiple vital signs in a single device.

### 4.4. Limitations

The limitations of this scoping review include the variation in and lack of data available within the selected articles. Only eight articles fit the inclusion criteria described, reflecting the early stage and underdeveloped research on newborn wireless monitoring technologies in the delivery room. The small number of articles and technologies available limits the generalizability of the findings and prevents an expansive conclusion on the effectiveness and benefits of wireless vital sign monitoring in the delivery room and specific patient outcomes. It also excluded devices that were used outside of the delivery room setting, for example, the NICU, which may have the potential to be used for monitoring after birth. Regardless, the goal of this scoping review was to evaluate the current availability of wireless monitoring technologies for newborns in the delivery room and ultimately provided a valuable summary of the current state of research in this setting.

## 5. Conclusions

This scoping review identified a growing number of wireless technologies for monitoring newborns immediately after birth. However, these technologies vary considerably in their stage of development and clinical readiness. Some wireless wearable systems, particularly ECG-based devices such as NeoBeat, have been evaluated across multiple clinical studies and are approaching broader implementation in delivery room care. It is worth mentioning that there are currently four ongoing clinical trials not included in this scoping review (Figure 1). In contrast, several emerging wearable systems and non-contact technologies have only been assessed in limited-feasibility or proof-of-concept studies and require further validation before routine clinical adoption.

Overall, the evidence base remains characterized by small sample sizes, short monitoring periods, and limited assessment of safety and long-term performance. Future research should focus on large-scale clinical validation, standardized evaluation methodologies, and the development of integrated systems capable of accurately monitoring multiple physiological parameters in the unique delivery room environment.

## Figures and Tables

**Table 1:** Methodological components of included papers.

Included Articles	*n* = 8	References
Study site *:	*n* (%)	
Delivery room	8 (100)	[22,23,24,25,26,27,28,29]
Age of participants *:	*n* (%)	
Term infants	8 (100)	[23,24,25,26,27,28,29]
Preterm infants	5 (62.5)	[22,23,25,28,29]
Low-birthweight infants (<2500)	5 (62.5)	[22,23,25,28,29]
Very low-birthweight infants (<1500)	1(12.5)	[22]
Duration of monitoring *:	*n* (%)	
<10 min.	4 (50)	[22,23,28,29]
>10 min, <1 h	2 (25)	[24,26]
>1 h	1 (12.5)	[27]
Unspecified	1 (12.5)	[25]
Type of statistical analysis	*n* (%)	
Bland–Altman	5 (62.5)	[22,24,26,27,29]
Correlation coefficient (*r*)	5 (62.5)	[22,23,26,27,28]
Basic descriptives	8 (100)	[22,23,24,25,26,27,28,29]
Outcomes reported	*n* (%)	
Safety	2 (25)	[23,28]
Feasibility	7 (87.5)	[22,23,24,25,26,28]
Accuracy	7 (87.5)	[22,23,24,26,27,28,29]

* Data are presented as *n* (%).

**Table 2:** Summary of devices.

Device Name	Vital Signs Monitored	Infants	Type of Technology	ROI	Vital Signs Computed in Real Time or Offline	Data Transfer Method	References
NeoBeat	Heart Rate	Preterm infants Term infants LBWI VLBWI	Wearable	Torso	Real time	Bluetooth	[22,23,28,29]
SurePulse VS (Nottingham, UK)	Heart Rate, Oxygen Saturation	Preterm infants Term infants LBWI	Wearable	Head	Real time	Bluetooth	[24,25,26]
ComfTech HOWDY Jacket (Monza, Italy)	Heart Rate	Term infants	Wearable	Torso	Real time	Wi-Fi	[28]

Legend: ROI = region of interest, LBWI = low-birthweight infant (<2500 g), VLBWI = very low-birthweight infant (<1500 g).

**Table 3:** Number and type of signals reported for included devices.

Included Articles	*n* = 8	References
Number of signals reported	*n* (%)	
1	7 (87.5)	[22,23,24,25,27,28,29]
2	1 (12.5)	[26]
3	0	
Number of vital signs reported	*n* (%)	
1	7 (87.5)	[22,23,24,25,27,28,29]
2	1 (12.5)	[26]
3	0	
Vital signs	*n* (%)	
Heart rate	8 (100)	[22,23,24,25,26,27,28,29]
Temperature	0	
Oxygenation saturation	1 (12.5)	[26]
Method used to obtain HR	*n* (%)	
Reflectance photoplethysmography (PPG)	3 (37.5)	[24,25,26]
Electrocardiography (ECG)	5 (62.5)	[22,23,27,28,29]
Non-contact electric-potential (EPS)	0	
Method used to obtain SpO2	*n* (%)	
Pulse oximeter (PPG-based)	1 (12.5)	[26]
Near-infrared spectroscopy	0	
Type of wireless information transfer:	*n* (%)	
Bluetooth	7 (87.5)	[22,23,24,25,26,28,29]
Wi-Fi	1 (12.5)	[27]
No wireless information transfer	0	
Not specified	0	

**Table 4:** Location and method of placement for included devices.

Included Articles	*n* (%)	References
Means of securing device:	8 (100)	
Band OR elastic OR strap	0	
Adhesive	0	
Garment	4 (50)	[24,25,26,27]
Non-contact	0	
Not secured	4 (50)	[22,23,28,29]
Device placement on body:	8 (100)	
Head	3 (37.5)	[24,25,26]
Neck	0	
Torso	5 (62.5)	[22,23,27,28,29]
Upper limb	0	
Lower limb	0	
Not on body	0	
Heart rate monitoring device placement:	8 (100)	
Head	3 (37.5)	[24,25,26]
Torso	5 (62.5)	[22,23,27,28,29]
Upper limb	0	
Lower limb	0	
Oxygen saturation monitoring device placement:	1 (100)	
Head	1 (100)	[26]
Upper limb	0	
Lower limb	0	

In certain cases, more than one site on the body was used for vital monitoring; thus, each category included was counted.

## Data Availability

Data will be made available upon request to the authors due to the file being to large to upload at Supplementary Materials.

## Supplementary Materials

The following supporting information can be downloaded at: https://www.mdpi.com/article/10.3390/children13081025/s1, Document S1: Data extraction form; Table S1: Search strategy key terms; Table S2: Exclusion and inclusion criteria for articles; Table S3: Name of journals included in review.

## Abbreviations

The following abbreviations are used in this manuscript:

ECG	Electrocardiography
IQR	Interquartile Range
MIN	Minutes
HR	Heart Rate
SpO2	Oxygen Saturation
PPG	Photoplethysmography
NICU	Neonatal Intensive Care Unit
FIC	Family Integrated Care
SUPC	Sudden Unexpected Postnatal Collapse
