# Supplementary material for: Non-Contact and Wireless Wearable Technologies for Neonatal Vital Sign Monitoring in the Delivery Room—A Scoping Review
Source: Children (Basel). 2026 Aug 1;13(8):1025. doi: 10.3390/children13081025 (PMC13510506; doi:10.3390/children13081025)
Supplement: Supplementary file 1 [file children-13-01025-s001.zip › Supplementary Document S1.pdf]

Supplementary Document S1. Data Extraction Form

Section 1: General Information

**Title of paper**	
**Study DOI**	
**Year of publication**	
**Journal published in**	
**Author(s)**	
**Corresponding authors contact information**	
**Number of study sites**	
**Continent in which study was conducted**	
**Country in which study was conducted**	
**Study funding source**	
**Do the authors have any conflict of interest to declare?**	

Section 2: Study Characteristics

**Aim of the study**	
**Study Design**	
**Study location (i.e. Delivery Room, clinic, et…)**	
**Inclusion Criteria**	
**Exclusion Criteria**	
**Total number of participants**	
**Were any of the following groups of infants included in the study**	
**Preterm infants**	
**Term infants**	
**Very low birthweight infants (<1500)**	
**Low birthweight infants (<2500)**	
**Other relevant participant information:**	

Section 3: Study Methods

**Was a reference technology used?**	
**Name of the reference technology**	
**Was there a pre-defined recording duration**	
**What was pre-defined recording duration**	
**Recording duration (actual)**	

	**State group (i.e. all or subgroup)**	**Min recording period**	**Max recording period**	**Mean/median recording period**	**Variability in recordings**	**Total duration of recording**
**1**						
**2**						
**3**						

**Types of outcomes reported in the study:**	
Safety	
Feasibility	
Accuracy	
Clinical	
Other	

Provide brief description of what outcomes were assessed and their endpoints

**Outcome**	**Describe what was assessed**	**Endpoint**
Safety		
Feasibility		
Accuracy		
Clinical		
Other		

Section 4: Study Results

- Summarize the findings for each type of outcome reported.

Outcome	Main findings
**Safety**	
**Feasibility**	
**Accuracy**	
**Clinical**	
**Other**	

- Select any of the following reported for feasibility:
  - Basic descriptive measures (mean, median, mode, min, max, IQR, SD, variance)
  - Bland Altman
  - Mixed Effect Model
  - Correlation
  - Variation accounted For
  - Parametric significance testing
  - Non-parametric significant testing
  - Other:
- Values for any of the feasibility outcomes selected above.

- Select any of the following reported for safety:
  - Basic descriptive measures (mean, median, mode, min, max, IQR, SD, variance)
  - Bland Altman
  - Mixed Effect Model
  - Correlation
  - Variation accounted For
  - Parametric significance testing
  - Non-parametric significant testing
  - Other:
- Values for any of the safety outcomes selected above.

- Select any of the following reported for accuracy:
  - Basic descriptive measures (mean, median, mode, min, max, IQR, SD, variance)
  - Bland Altman
  - Mixed Effect Model
  - Correlation
  - Variation accounted For
  - Parametric significance testing
  - Non-parametric significant testing
  - Other:
- Values for any of the accuracy outcomes selected above.

Section 5 – Monitoring Technology Information
- Device name.
- Type of technology being tested (non-contact and/or wireless).

- For non-contact monitoring system please specify relevant equipment for non-contact recording.
- How many devices are placed on the infant in the study
- Where are wearables applied to the body OR if what is the ROI for non-contact technologies
    - Head
    - Neck
    - Torso
    - Upper limbs
    - Lower limbs
    - N/A (not specified)
- Provide specific wearable/ROI defined location.
- Were any modifications made to Delivery Room set up/care for study
- Were vital signs computed in real-time or offline.
- Which vital signs were monitored by the device.
- Which methods were used to monitor HR.
- Which methods were used to monitor RR.
- Which methods were used to monitor SpO2.
- Which methods were used to monitor temperature
- Additional information about vital signs monitored (i.e. lead configuration, averaging windows, etc…)

	**Additional Information**
**HR**	
**RR**	
**SpO2**	
**Temp**	
**Blood pressure**	
**Other**	

- Did the device monitor non-vital signs?
    - What non-vital signs were monitored

Section 6 – Study Quality Assessment

- Did the results include all participants data in analysis?
    - Yes
    - No
    - Unsure
- If selected “No” please indicate explanation/justification for data exclusions provided.
- If indicated provide number of hours/participants excluded from the study
- Does the selection process of patients introduce bias? Risk:
    - High
    - Low
    - Unclear
- Please provide justification if “High” or “Unclear” selected in previous question.
- Was the study population appropriate for review question? Concern:
    - High
    - Low
    - Unclear
- Please provide justification if “High” or “Unclear” selected in previous question.
- Does the conduct or the interpretation if the index test introduce bias? Risk
    - High
    - Low
    - Unclear
- Please provide justification if “High” or “Unclear” selected in previous question.
- Does the conduct or the interpretation of the index test differ from the review question? Concern:
    - High
    - Low
    - Unclear
- Please provide justification if “High” or “Unclear” selected in previous question.
- Does the conduct or the interpretation if the reference test introduce bias? Risk
    - High
    - Low
    - Unclear
- Please provide justification if “High” or “Unclear” selected in previous question.
- Does the conduct or the interpretation of the reference test differ from the review question? Concern:
    - High
    - Low
    - Unclear
- Please provide justification if “Low” or “Unclear” selected in previous question.
- Could the flow and timing of study procedures (reference and index) introduce bias? Concern:
    - High
    - Low
    - Unclear
- Concern for other sources of bias?
