# Supplementary material for: Non-Contact and Wireless Wearable Technologies for Neonatal Vital Sign Monitoring in the Delivery Room—A Scoping Review
Source: Children (Basel). 2026 Aug 1;13(8):1025. doi: 10.3390/children13081025 (PMC13510506; doi:10.3390/children13081025)
Supplement: Supplementary file 1 [file children-13-01025-s001.zip › Supplementary Table S1.pdf]

Supplementary Table S1. Search strategy key terms

1	exp infant, newborn/
2	(babe or babes or baby or babies or infant* or infantile or infancy or low birth weight or low birthweight or neonat* or neo-nat* or newborn* or new born* or newly born or premature or pre-mature or pre-matures or prematures or prematurity or pre-maturity or preterm or preterms or pre term or pre terms or preemie or preemies or premies or premie or VLBW or VLBWI or VLBW-I or VLBWs or LBW or LBWI or LBWs or ELBW or ELBWI or ELBWs).ti,ab,kf.
3	1 or 2
4	birthing centers/ or delivery rooms/
5	((birth* or delivery* or labor* or labour* or maternity or delivery or obstetric*) adj (center* or centre* or room* or suite* or ward* or unit or units or department or departments)).ti,ab,kf.
6	4 or 5
7	exp Monitoring, Physiologic/ or Electrocardiography/ or exp Vital Signs/ or exp Ultrasonography, Doppler/ or Photoplethysmography/
8	(electrocardiograph* or ultrasound* or ultrasonograph* or sonograph* or vital sign* or ((blood or diastolic or systolic) adj pressure*) or pulse or temperature* or ((heart or cardiac) adj2 (rate* or chronotrop*)) or heart beat* or heartbeat* or respiration* or respirator* or breathing or oxygen saturat* or plethysmograph*).ti,ab,kf.
9	7 or 8
10	Wireless Technology/ or forecasting/ or td.fs.
11	(wireless* or noncontact* or non contact* or "no contact" or contactless or "without contact" or noninvasive* or non invasive* or radar or laser* or video* or futurology or ((new or novel or innovat* or cutting edge or inventive* or creativ* or original* or imaginat* or leading edge or future or trend or trends or trending or emergent or emerging) adj3 (technolog* or sensor* or monitor*))).ti,ab,kf.
12	10 or 11
13	((ultrasound* or ultrasonograph* or sonograph*) and vital sign*).ti,ab,kf.
14	(3 and 9 and 12) or (3 and 13)
15	3 and 6 and 9
16	14 or 15
17	limit 16 to yr="2015 -Current"
