# Supplementary material for: Non-Contact and Wireless Wearable Technologies for Neonatal Vital Sign Monitoring in the Delivery Room—A Scoping Review
Source: Children (Basel). 2026 Aug 1;13(8):1025. doi: 10.3390/children13081025 (PMC13510506; doi:10.3390/children13081025)
Supplement: Supplementary file 1 [file children-13-01025-s001.zip › Supplementary Table S2.pdf]

Supplementary Table S2. Exclusion and Inclusion Criteria for Articles.

Inclusion	Exclusion
• Focus on newborns<br>• Monitoring done immediately after birth<br>• Any gender<br>• Any ethnicity<br>• Any pre-existing conditions<br>• Must be about monitoring devices<br>• Primary vital sign measurements (HR, RR, SpO2, temperature), and other clinically relevant physiological measurements<br>• Any geographic location<br>• No language restrictions<br>• Any peer reviewed original article or conference abstract/paper<br>• Published no earlier than 2015	• Studies focusing on the mother<br>• Animal and cadaver studies<br>• Monitors for the purpose of homecare, developmental monitoring, entertainment, primarily diagnostic testing devices<br>• Portable diagnostic testing devices<br>• Interventional wireless devices.<br>• Home monitoring studies<br>• Commercially advertised products without peer reviewed scientific evidence in support<br>• Research focused exclusively on device design<br>• Articles providing insufficient information on device<br>• Non-original studies<br>• Non-primary source studies (i.e., without primary data)
