# Supplementary material for: Non-Contact and Wireless Wearable Technologies for Neonatal Vital Sign Monitoring in the Delivery Room—A Scoping Review
Source: Children (Basel). 2026 Aug 1;13(8):1025. doi: 10.3390/children13081025 (PMC13510506; doi:10.3390/children13081025)
Supplement: Supplementary file 1 [file children-13-01025-s001.zip › Supplementary Table S3.pdf]

Supplementary Table S3. Name of Journals Included in Review

**Journal**	**Number of included articles**
Resuscitation	1
Acta Paediatrica	3
Archives of Disease in Childhood – Fetal and Neonatal Edition	1
Children	1
Frontiers in Pediatrics	1
Pediatric Research - Nature	1

Legend: Name of journal in which each included study was published, and number of studies included published in journal.
